# Reactive surveillance of suicides during the COVID-19 epidemic in France, 2020- March 2022

**DOI:** 10.1093/eurpub/ckac129.748

**Published:** 2022-10-25

**Authors:** A Fouillet, D Martin, I Pontais, C Caserio-Schönemann, G Rey

**Affiliations:** 1 Division of Data Sciences, Santé Publique France, Saint-Maurice, France; 2 CépiDc, Inserm, Kremlin-Bicêtre, France

## Abstract

**Background:**

Mitigation actions during the COVID-19 pandemic, in particular lockdowns and curfews, may impact mental health and suicide in general populations. We aimed to analyse the evolution in suicide deaths from January 2020 to March 2022 in France.

**Methods:**

Using free-text medical causes in death certificates, we built an algorithm, which aimed to identify suicide deaths. We measured its retrospective performances by comparing suicide deaths identified using the algorithm with deaths which had either an ICD10 code for ‘intentional self-harm’ or for ‘external cause of undetermined intent’ as underlying cause. The number of suicide deaths from January 2020 to November 2021 was then compared with the expected number estimated using a generalized additive model. The analysis was stratified by age group and gender. Analysis from December 2021 to March 2022 was conducted using electronic death certificates only.

**Results:**

The free-text algorithm demonstrated high performances. From January 2020 to November 2021, suicide mortality declined during France's three lockdowns, particularly in men, and remained quite comparable with expected values between and after both of the country's lockdowns. Provisional results based on electronic death certificates suggest that suicide mortality remained stable until March 2022.

**Conclusions:**

Monitoring suicide mortality is possible in France with a 4-month delay; this will be reduced to two days when electronic death certification is fully deployed. This study highlighted the absence of an increase in suicide mortality during France's COVID-19 pandemic, and a substantial decline during lockdowns periods, something already observed in other countries. Further studies are required to explain the factors for this decline.

**Key messages:**

• In the absence of reactive coding of medical causes of deaths, the study proposed an approach to reactively identify suicide based on free-text medical causes from death certificates.

• Our findings provide reassurance that the COVID-19 pandemic has not had a negative impact on the general population in terms of suicide in France from March 2020 to September 2021.

